# Hepatic Runx1t1 improves body fat index after endurance exercise in obese mice

**DOI:** 10.1038/s41598-023-46302-w

**Published:** 2023-11-08

**Authors:** Ning Jiang, Zhe Wang, Xiangying Guo, Zifu Peng, Yimin He, Qian Wang, Huaduo Wu, Yunlong Cui

**Affiliations:** 1 Tianjin Key Laboratory of Exercise Physiology and Sports Medicine, Institute of Sport, Exercise and Health, Tianjin University of Sport, Tianjin, 301617 China; 2 Department of Basic Teaching of Military Common Subjects, Logistics University of Chinese People’s Armed Police Force, Tianjin, 300309 China; 3 Department of Hepatobiliary Surgery, Tianjin Medical University Cancer Institute and Hospital, Huanhu West Road, Hexi District, Tianjin, 300061 China

**Keywords:** Computational biology and bioinformatics, Animal physiology, Genetics research, Biomarkers

## Abstract

Endurance exercise could attenuate obesity induced by high fat diet (HFD). Thus, the purpose of this study was to explore the crucial targets that play key roles in the improvement of body fat index (BFI) in obese mice by endurance exercise. Firstly, we constructed murine obesity models: High fat diet control (HFD) group, HFD exercise (HFE) group, normal chow diet control (NC) group, and normal chow diet exercise (NE) group. Next, we identified the BFI improvement related genes using differential gene analysis, and investigated these genes’ functional pathways using functional enrichment analysis. The qRT-PCR and western blot assays were used to determine the gene expression and protein expression, respectively. Gene set enrichment analysis was used to explore the potential pathways associated with endurance exercise in obese mice and Mitochondrial respiratory control ratio (RCR) assay was applied to determine the RCR in the liver tissues of mice. We discovered that endurance exercise remarkably reduced the body weights and BFI of HFD-induced obese mice. *Runx1t1* was related to the improvement of BFI by endurance exercise in HFD-induced obese mice. *Runx1t1* mRNA and protein levels in liver tissues were observably decreased in HFD mice compared to mice in HFE, NC and NE groups. Moreover, Glucagon signaling pathway that was associated with mitochondrial function was significantly activated in HFE mice. The *Runx1t1* expression exhibited an observable negative correlation with *Acaca* in HFD mice. Moreover, the mitochondrial RCR level was significantly increased in HFE mice than that in HFD mice. In HFD-induced obese mice, *Runx1t1* was implicated in the improvement of BFI via endurance exercise. Endurance exercise could improve mitochondrial dysfunction in obese mice by activating the *Runx1t1*.

## Introduction

Obesity is a multifactorial disease characterized by excess body fat that may be associated with altered nutritional status, secondary to genetic, iatrogenic or endocrine disorders^1^. Obesity has become a growing global public health problem over the past few decades^2^. It has been reported that obesity remarkably increases the risk of many chronic diseases, such as type II diabetes, cardiovascular disease, non-alcoholic fatty liver disease, etc.^3^. Obesity is more prevalent in developed countries, but has increased dramatically in developing countries in recent years^4^. In addition, the prevalence of obesity has improved dramatically among children and adolescents in both developed and developing countries, causing detrimental effects on their physical and mental health^5^.

The liver is an important hub for many physiological processes, including nutrient metabolism, blood volume regulation, immune system support, endocrine control of growth signaling pathways, lipid and cholesterol homeostasis and the breakdown of exogenous compounds^6^. Nonalcoholic fatty liver disease (NAFLD) refers to excessive hepatic fat accumulation in the absence of secondary hepatic steatosis, relating to a high intake of alcohol or other relevant causes^7^. The prevalence of NAFLD is increasing with the epidemic of obesity and type II diabetes worldwide^8^. Furthermore, NAFLD is also a multisystem disorder associated with type II diabetes, cardiovascular disease, and chronic kidney diseases^9^. Many factors, such as genetic factors, obesity, dietary intake, physical inactivity, and insulin resistance, can contribute to the development and progression of NAFLD^10,11^. Thus, considering the current epidemic of obesity and the increased risk of chronic diseases caused by obesity, there is an urgent need for more novel therapeutic strategies to help people prevent/reverse the onset of obesity.

Exercise is an excellent tool for the prevention and adjunctive treatment of metabolic diseases^12^. As a metabolically active organ, liver has many functions related to metabolism and digestion. The liver is able to oxidize lipids, meanwhile it could also pack excess lipids to store them in other tissues, such as fat^6^. As physical inactivity leads to hypertrophy of visceral adipose tissue, it has been universally accepted that sedentary lifestyle would trigger systemic inflammation and increase the risk of chronic diseases^13^. Exercise is considered a viable therapeutic strategy for the treatment of obesity to optimize body composition, especially as an adjunct to bariatric surgery^14^. Accumulating evidences have suggested that endurance exercise can alleviate immunometabolic tissue disorders in obesity^15^. In addition, endurance exercise mediates the repair of metabolic and redox imbalances and plays an important role in preventing or delaying the severe progression of NAFLD^16^. A recent study has indicated that moderate endurance exercise improves skeletal muscle metabolic health and increases muscle adiponectin production in hyperlipidemic mice^17^. Melo et al. have found that endurance exercise alters hepatic gene expression and signaling pathways associated with metabolic disease in male and female mice^18^. However, as far as we know, there is limited exploration of the important hepatic genes related to the improvement of body fat index by endurance exercise in obese mice. Thus, this study aimed to explore the targets that play key roles in the improvement of body fat index in obese mice by endurance exercise, providing more transcriptome level information for the improvement of body fat index by endurance exercise.

## Study subjects

### Mice

Seven-week-male C57BL/6 mice (n = 48) with body weight of 20 ± 1 g were provided by Beijing vitong Lihua Laboratory Animal Technology Co., Ltd. (Beijing, China). All mice were housed in specific-pathogen-free facilities (four animals per cage) with light/ dark cycle for 12 h and room temperature maintained at 22 ~ 25 °C, and they were provided with free access to food and water during the housing. The laboratory mice were euthanized by cervical dislocation. The present study was approved by the Medical Ethics Committee of Tianjin University of Sport (nos. TIUS2320-010). All animal experiments in this study were conducted according to the institutional guidelines of the Animal Care and Use Committee at Tianjin University of Sport. This study is reported in accordance with ARRIVE guidelines (https://arriveguidelines.org).

### Study design

Forty-eight mice were subjected to adaptive feeding with regular chow diet feeds for 1 week, and then randomly divided into two groups: normal chow diet (NCD, n = 16) and high fat diet (HFD, n = 32) groups. The mice in NCD group were fed with normal chow diet (including ≤ 10.0% moisture, ≥ 18% crude protein, ≥ 4.0% crude fat, ≤ 5.0% crude fiber, ≤ 8.0% crude ash, 1.0 ~ 1.8% calcium and 0.6 ~ 1.2% phosphorus, Beijing huafukang Bioscience Co., Ltd) and in HFD group were fed with high fat diet (20% kcal from protein, 60% kcal from fat, 20% kcal from carbohydrate, Beijing Huafukang Bioscience Co., Ltd, Beijing, China) for 12 weeks. The body weight of mice was measured and recorded during the feeding period, the bedding material was changed and drinking water was added at least once a week. After 12 weeks, the body weights of 16 mice in HFD group exceeded 20% of the mean body weights of mice in the NCD group. Subsequently, The NCD mice were randomly divided into two groups: NCD control (NC) and NCD exercise (NE) groups. The HFD mice were spilt into HFD control (HFD) group and HFD exercise (HFE) group. The mice in NC and HFD groups did not undergo endurance exercise training, and the mice in NE and HFE groups underwent an 8-week endurance exercise training (treadmill).

One week before the main exercise training program, mice in the NE and HFE groups were subjected to pre-exercise training session, during which the training duration and speed were gradually increased (from 8 m/min exercise for 20 min to 10 m/min exercise for 50 min).

For exercise training, the mice exercised on a treadmill for 60 min at an intensity consisting of 10 m/min (10 min), 12 m/min (40 min) and 10 m/min (10 min) at 0% grade for 1 to 4 weeks. During weeks 5–8, the exercise rate increased to 10 m/min (10 min), 14 m/min (40 min), and 10 m/min (10 min) at the same slope for training, 60 min/session, 5 sessions/week and the mice had a rest on Saturday. The mouse body weight and food intake were recorded weekly during the exercise intervention. After the end of the intervention, one mouse was dead in the NE and HFE groups by accident, respectively.

### Sample preparation

After last training session 24 h, the mice in the exercise groups (NE, HFE) and non-exercise groups (NC, HFD) were fasted for 12 h, and weighed their body weights after 24 h. The liver samples were collected in cryovials and frozen in liquid nitrogen and stored at -80 °C. The epididymal and perirenal adipose tissues of mice were harvested and washed with pre-chilled PBS and weighed, which was used to calculate the body fat index (BFI).

$${\text{BFI}} = \left( {{\text{left}}\;{\text{and}}\;{\text{right}}\;{\text{perirenal}}\;{\text{fat}} + {\text{right}}\;{\text{and}}\;{\text{left}}\;{\text{epididymal}}\;{\text{fat}}} \right)/{\text{body}}\;{\text{weight}} \times {1}00\%$$

### mRNA transcriptome sequencing

Next, 1 μg total RNA was taken for library preparation, poly (A) mRNA was isolated using 0ligo (DT) beads, synthesized first-strand cDNA and the second-strand cDNA and repair, and add a dA-tailing, followed by a T-A ligation to add adaptors to both ends. Each sample was then amplified by PCR using P5 and P7 primers. The sequencing was enforced using a 2 × 150 paired-end (PE) configuration on an Illumina HiSeq/Illumina Novaseq/MGI2000 instrument.

### Data processing and differential gene expression analysis

Trimmomatic was used to filter all sample data, filtered data were aligned to the reference genome (GRCm39) using Hisat2. The count value of each gene in the sample was calculated using the software StringTie, and the value was used to perform the differential significance analysis of all genes in the samples of each group to find the relative differentially expressed genes (DEGs). The DEGs were screened using the DESeq2 function package in R language (version 4.1.0, the same below), according to |log_2_FC|> 1 and *P* < 0.05.

### Function enriched analysis

The DEGs were subjected to the enrichment analysis, including Gene ontology (GO, Biological Process (BP), Molecular Function (MF) and Cellular Component (CC)) and Kyoto Encyclopedia of Genes and Genomes (KEGG) pathway, using “clusterProfiler”^19^ in R language. The *P* < 0.05 was applied to screen the significantly enriched pathways.

### Gene set enrichment analysis (GSEA)

The GSEA was conducted employing “clusterProfiler” package in R language, and the significantly enriched KEGG pathways were screened by |NES|> 1 and *P* < 0.05.

### qRT-PCR

In NC, NE, HFD, HFE groups, we randomly selected three mice, respectively, and collected their liver tissues. Total RNA from liver tissues were extracted using TRNzol Universal total RNA Extraction Reagent (DP424, Tiangen, Beijing, China). The extracted RNA was quantified and its purity was checked using an ultramicro spectrophotometer (Nanodrop2000, Thermofisher, New York, USA). Subsequently, the cDNA was synthesized using the RevertAid First Strand cDNA Synthesis Kit (K1622, Thermo Fisher, New York, USA) on a PCR machine (Veriti, ABI, Thermo Fisher, New York, USA) and was subjected to fluorescent quantitative PCR on a real time PCR machine (Step One Plus, ABI, Thermo Fisher, New York, USA) using 2 × SYBR Green qPCR Master Mix (Servicebio, Wuhan, China) according to the manufacturer’s protocol. The primer sequences were displayed in Table 1, and the internal reference was *GAPDH*. mRNA expression levels were determined with 2^−ΔΔCT^ formula.

**Table 1:** Primer sequences for qRT-PCR.

Genes	Forward Primer (5′-3′)	Reverse Primer (5′-3′)	Product length (bp)	Tm (°C)
GAPDH	GAAGGTGAAGGTCGGAGTC	GAAGATGGTGATGGGATTTC	227	58
Runx1t1	GCCAGCGGTACAGTCCAAATAAT	CTGCCCATTCTCTGTCTGTTAGT	237	61

### Western blot

The liver tissues (0.1 g per sample) of mice were lysed in RIPA buffer (R002, Beijing Solarbio Science & Technology Co., Ltd, Beijing, China) to obtain protein samples. The protein concentration was determined by BCA protein concentration assay kit (P0011, Beyotime, Shanghai, China). Next, the proteins were separated by sodium dodecyl sulfate polyacrylamide gel electrophoresis (SDS-PAGE). The separated proteins were transferred onto polyvinylidene fluoride (PVDF) membranes (Ipvh00010, Merck biologicals, Darmstadt, Germany) in tris–glycine transfer buffer for 30 min. Membranes were blocked in 5% nonfat dry milk (P2016-1500 g, Beyotime, Shanghai, China) for 2 h at room temperature and incubated overnight at 4 °C with primary antibody RUNX1T1 Polyclonal Antibody (PA5-79943, 1: 1000, ThermoFisher, NewYork, USA). Subsequently, the membranes were washed with Tris buffered saline with 0.1% Tween20 (TBST) and incubated for 1 h with secondary antibody sheep anti-rabbit IgG HRP (bs-0295G-HRP, 1: 3000, Bioss, Beijing, China). Finally, the membranes were washed with TBST and placed in ELC chromogenic reagents (P0019M, Beyotime, Shanghai, China) for 2 min and imaged and photographed by a fully automated chemiluminescence image analysis system (Tanon5200, Shanghai, China). The internal reference used was GAPDH (UM4002, youanti, Tianjin, China). The gray values of banks were analyzed using the software Gelpro32.

### Mitochondrial respiratory control ratio (RCR) assay

Then fresh liver tissues (50 mg per sample) were broken and placed in a glass homogenizer, which were centrifuged to collect the supernatant. The supernatant was resuspended by adding 2 mL mitochondrial separation medium, and the pellet was collected by centrifugation. The state 3 respiration and state 4 respiration of extracted mouse liver mitochondria were measured using an OROBOROS Oxygraph-2 k high-resolution respirometry system (Oroboros Co, Austria), and the respiratory control rate (the ratio of state III respiration rate to state IV respiration rate) was calculated to evaluate the respiratory function of liver mitochondria.

### Statistical analysis

All bioinformatics data were analyzed using R software (Version 4.2.0). The difference among various groups was determined by Wilcoxon rank-sum test. All experimental data were presented as mean ± standard deviation (SD). Data were analyzed by one-way ANOVA and two-way ANOVA using SPSS 25.0 software. The *p* < 0.05 was considered statistically significant.

### Ethical approval

The present study was approved by the Medical Ethics Committee of Tianjin University of Sport (nos. TIUS2320-010). All animal experiments in this study were conducted according to the ethical guidelines of the Animal Care and Use Committee at Tianjin University of Sport. The study was in accordance with the ARRIVE guidelines.

## Results

### Endurance exercise reduced BFI in HFD-induced obese mice

**Figure 1:**
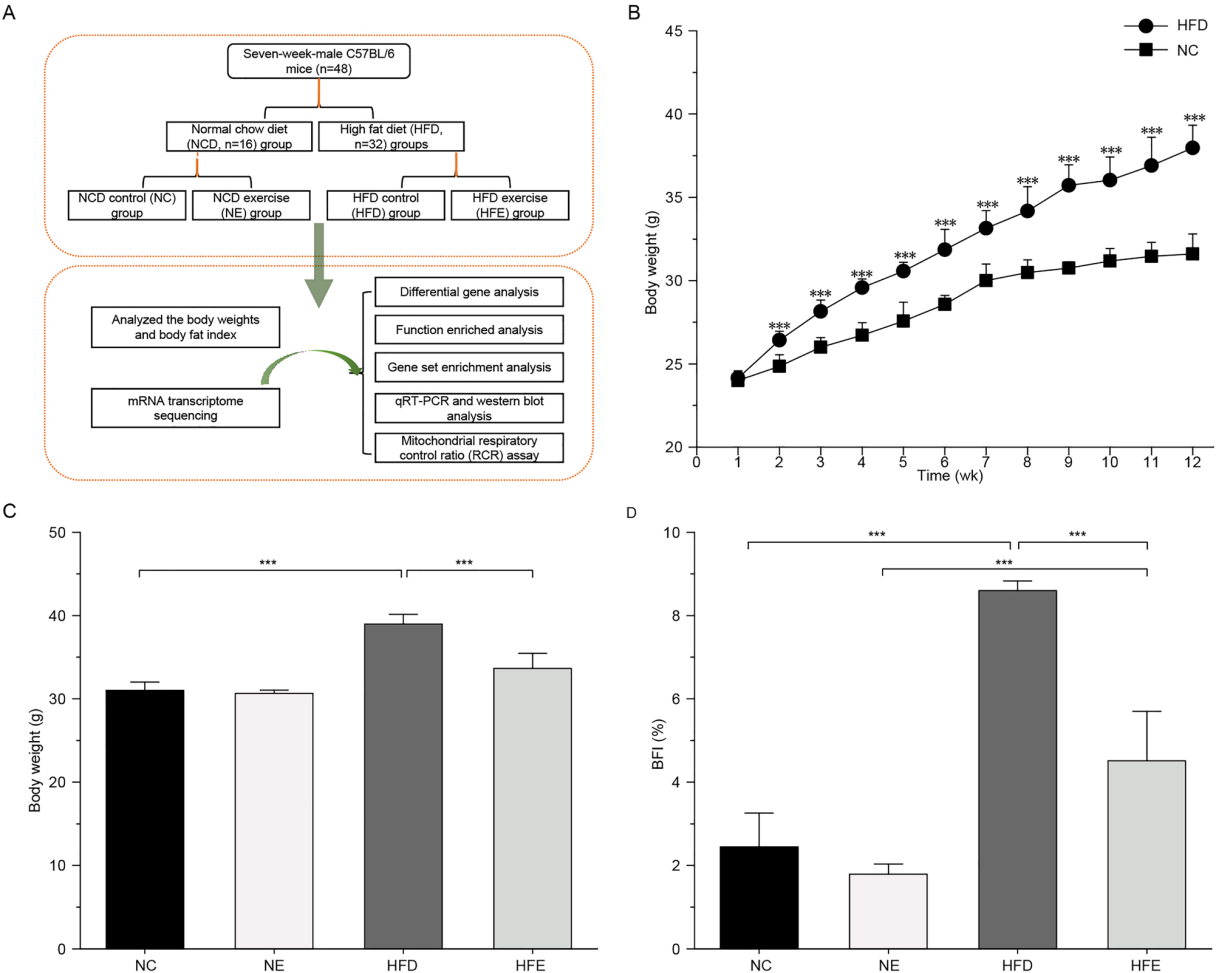
Endurance exercise reduces body fat index in obese mice. (**A**) The flow chart of this work. (**B**) The effect of high fat diet on the body weight of mice. (**C**) Effect of endurance exercise on body weight of mice. (**D**) Effect of endurance exercise on BFI of mice. ***P < 0.001,

To explore the crucial targets that play key roles in the improvement of obese mice by endurance exercise, obese mouse models were built, and the flow chart of our work was displayed in Fig. 1A. The body weight of mice showed no significant difference between NC (22.62 ± 0.59 g) and HFD (23.06 ± 0.82 g) groups before dietary intervention. After 2 weeks of feeding, the body weights of mice were significantly increased in HFD group than that in NC group, and the body weights of mice in NC and HFD were 31.31 ± 1.49 g and 33.58 ± 2.86 g fed for 12 h, respectively (Fig. 1B). Comparing to HFD group, the body weight and BFI of mice in HFE group was remarkably decreased (Fig. 1C,D). These results showed that endurance exercise could reduce the BFI of obese mice.

### Identification of genes related to the improvement of BFI by endurance exercise in HFD-induced obese mice

**Figure 2:**
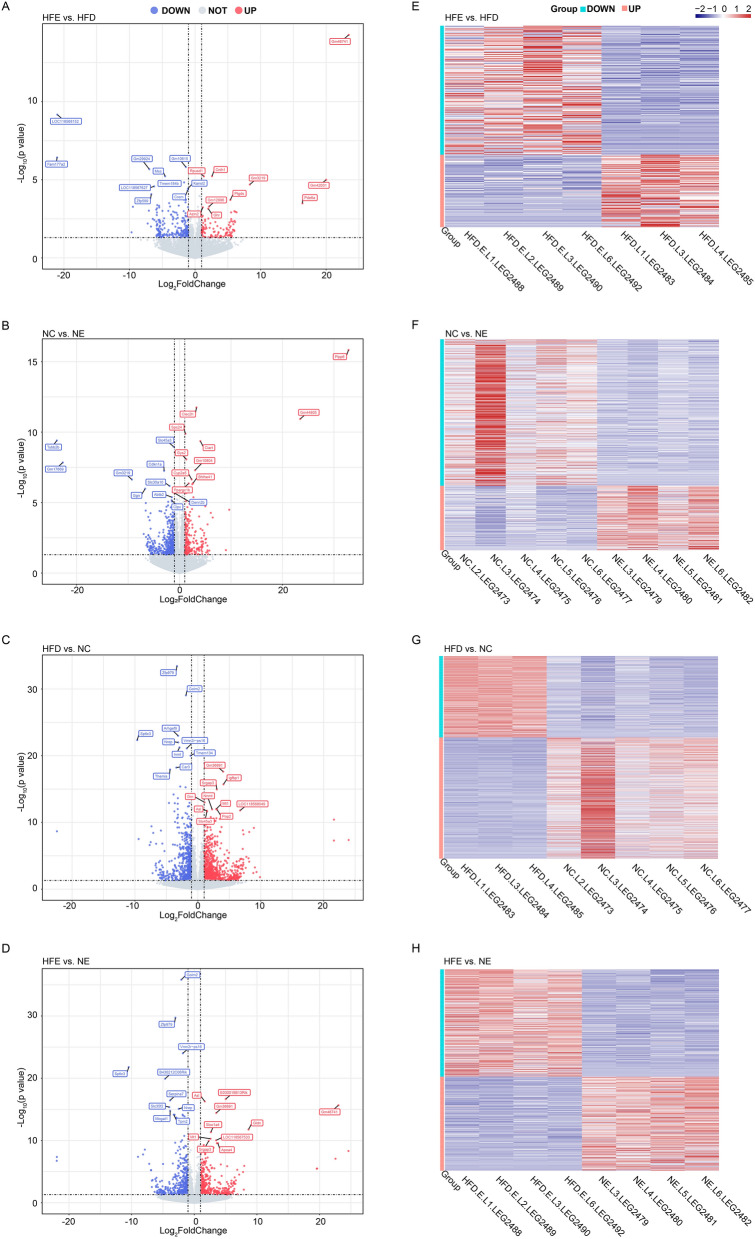
Identification of genes associated with the improvement of BFI in obese mice by endurance exercise. (**A**–**D**) The DEGs between HFE and HFD groups, NC and NE groups, HFD and NC groups, HFE and NE groups. (**E**–**H**) Heatmap of DEGs between HFE and HFD groups, NC and NE groups, HFD and NC groups, HFE and NE groups.

We performed mRNA transcriptome sequencing analysis of liver tissues from mice in NC, NE, HFD, HFE groups and obtained transcriptome sequencing data. Subsequently, we analyzed the DEGs between HFE and HFD groups, NC and NE groups, HFD and NC groups, HFE and NE groups, respectively. Totally 328 DEGs (including 118 up-regulated and 210 down-regulated genes (HFE vs. HFD)), 760 DEGs (including 232 up-regulated and 528 down-regulated genes (NC vs. NE)), 1636 DEGs (including 979 up-regulated and 657 down-regulated genes (HFD vs. NC)), and 842 DEGs (including 393 up-regulated and 449 down-regulated genes (HFE vs. NE)) were identified between HFE and HFD groups, NC and NE groups, HFD and NC groups, HFE and NE groups, respectively (Fig. 2A–D). In addition, the expressions of DEGs were significantly between HFE and HFD groups, NC and NE groups, HFD and NC groups, HFE and NE groups (Fig. 2E–H).

### *Runx1t1* played an important role in improvement of BFI by endurance exercise in HFD-induced obese mice

**Figure 3:**
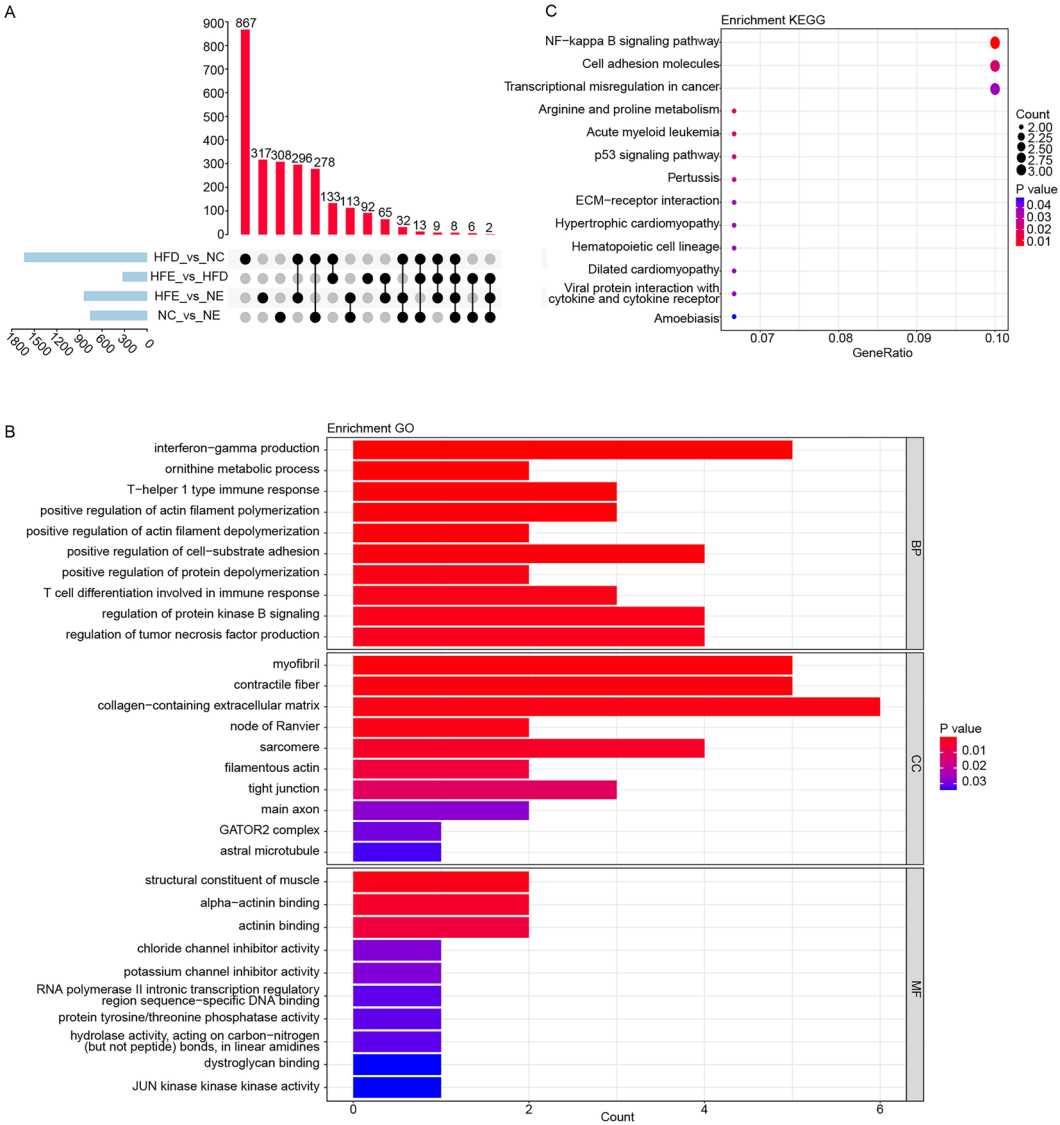
*Runx1t1* may an important gene in regulating fat metabolism in obese mice. (**A**) The setup diagram among four DEGs sets. (**B**) The top 10 significantly enriched GO terms (including BP, MF, CC). (**C**) The significantly enriched KEGG pathways.

**Figure 4:**
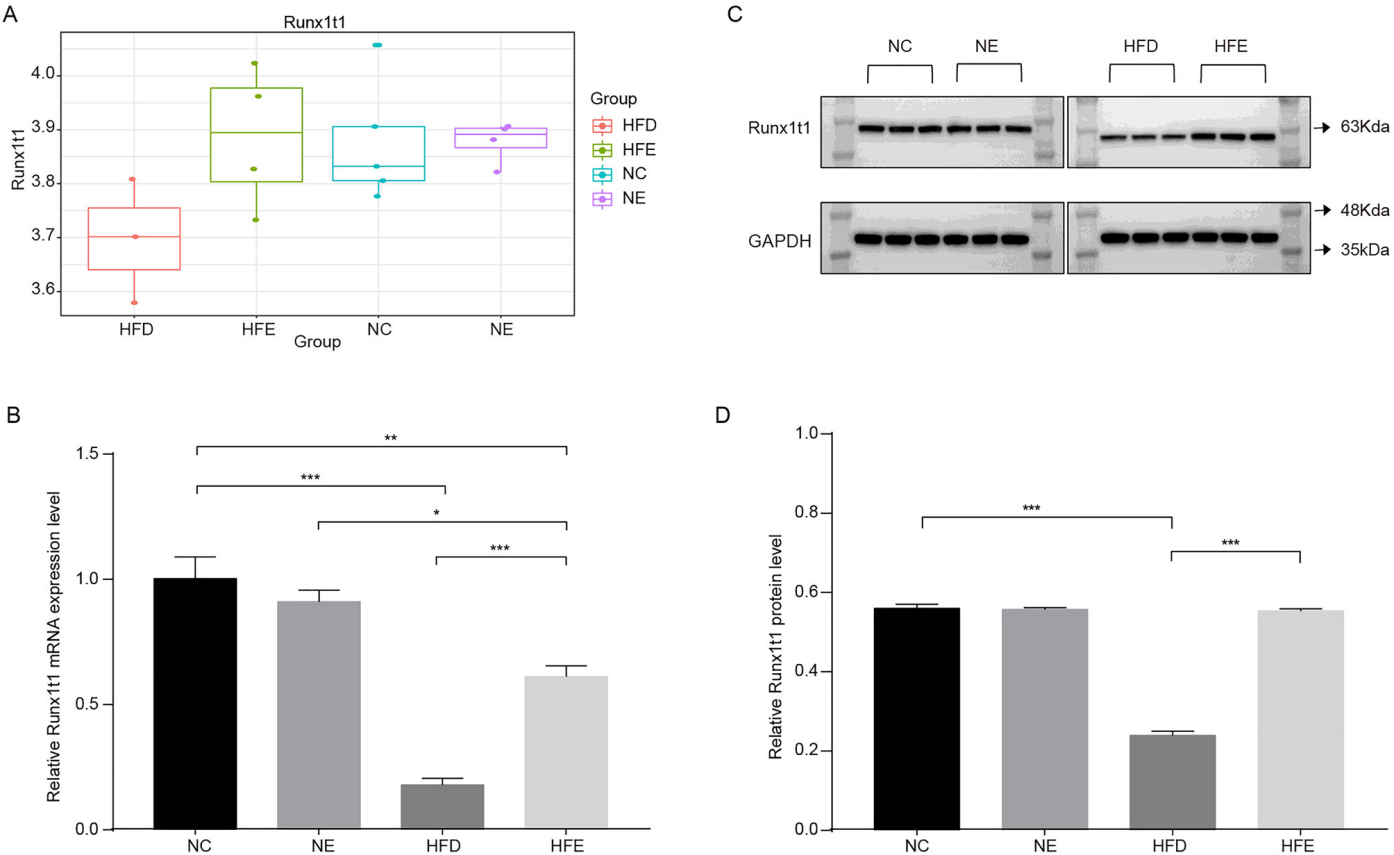
*Runx1t1* was down-regulated in obese mice. (**A**) The expression of *Runx1t1*. (**B**–**D**) The mRNA and protein levels of Runx1t1 in liver tissues of mice. **P < 0.01, ***P < 0.001.

To further precisely screen the key genes in endurance exercise improving obese mice, we analyzed the overlapping DEGs between HFE vs. HFD group and HFD vs. NC group, and removed the overlapping DEGs between NC vs. NE group and HFE vs. NE group, and eventually obtained 133 overlapping DEGs (Fig. 3A). These 133 genes were significantly enriched in 299 GO terms (Table S1, Fig. 3B), such as interferon-gamma production, myofibril, structural constituent of muscle and 13 KEGG pathways (Fig. 3C, Table S2). We found that 16 genes were significantly enriched in these 13 significant KEGG pathways among the 133 genes (Table S2). Of these 16 genes, *Runx1t1* was found to be a negative regulator of adipogenesis^20^. Subsequently, we analyzed the expression of *Runx1t1*, and found that *Runx1t1* was down-regulated in HFD group compared to HFE, NC and NE groups (Fig. 4A). To verify this result, we analyzed the levels of *Runx1t1* mRNA and protein in liver tissues of mice. As shown in Fig. 4B–D, the levels of Runx1t1 mRNA and protein expressions were not significantly differential between NC and NE group. However, comparing to HFD group, the levels of Runx1t1 mRNA and protein expressions were remarkably increased in HFE group. These results indicated that the endurance exercise might increase the levels of Runx1t1 mRNA and protein in obese mice, but not in normal mice.

### The potential pathways associating with endurance exercise in obese mice

**Figure 5:**
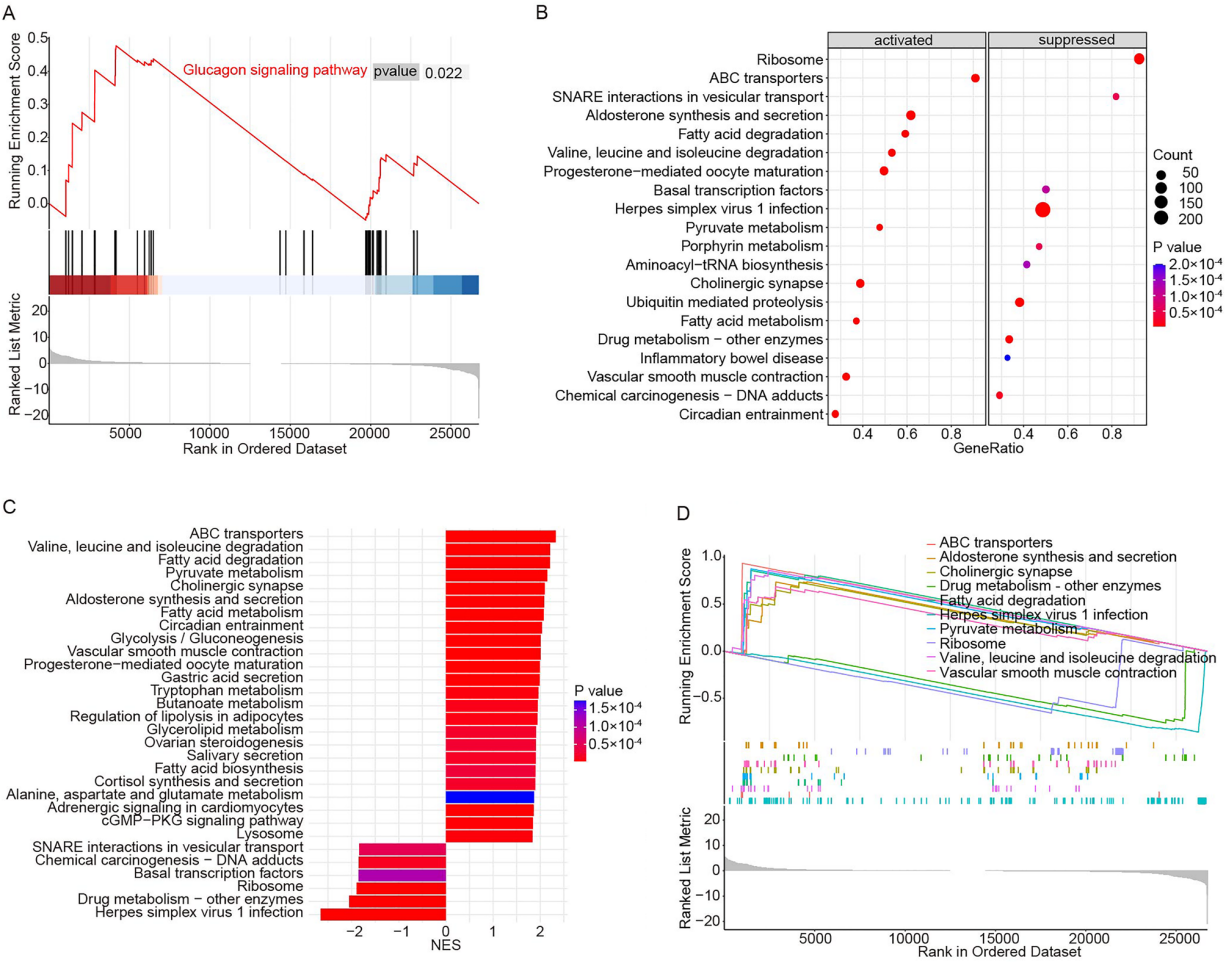
The potential pathways associated to endurance exercise in obese mice. (**A**) Glucagon signaling pathway. (**B**) The top 10 significantly activated or suppressed pathways in HFE group. (**C**, **D**) The top 30 and 10 significantly enriched pathways in HFE group.

The GSEA results showed that totally 103 and 41 pathways were remarkably activated and suppressed in HFE group compared to HFD group, respectively (Table S3). Of them, we noticed that glucagon signaling pathway was correlated with mitochondrial function (Fig. 5A). The top 10 significantly activated or suppressed pathways were displayed in Fig. 5B. The top 30 and 10 significantly enriched pathways were presented in Fig. 5C and D, respectively.

### Endurance exercise improved mitochondrial respiratory dysfunction in HFD-induced obese mice

**Figure 6:**
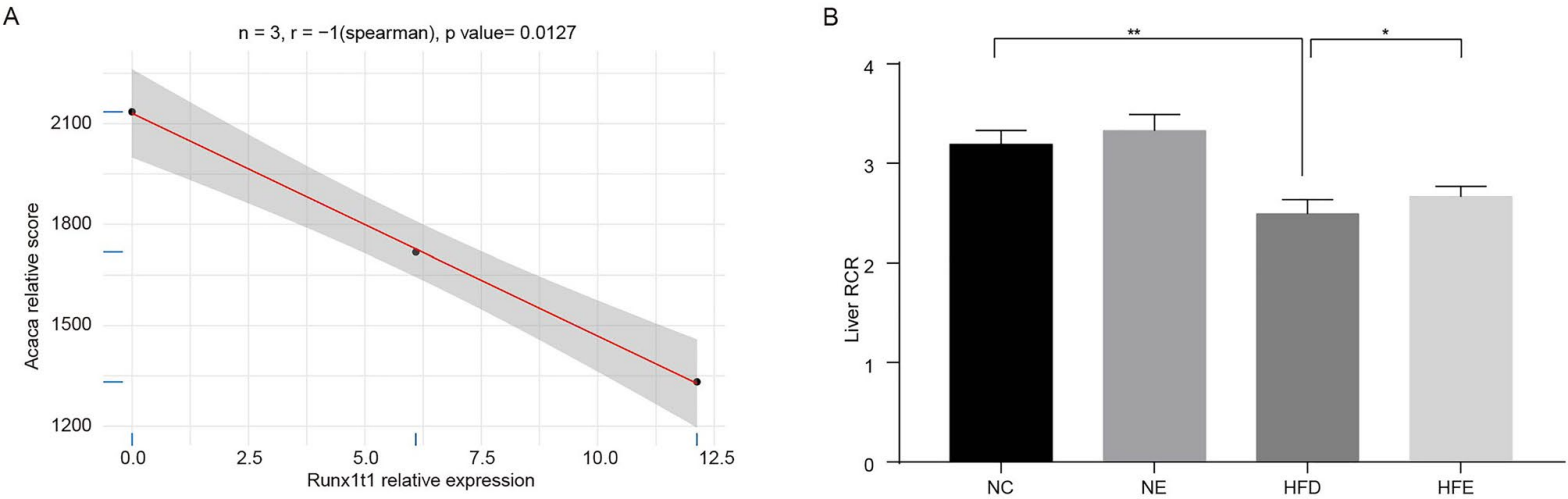
Endurance exercise improved mitochondrial respiratory dysfunction in obese mice. (**A**) The correlation of *Runx1t1* with genes significantly enriched in Glucagon signaling pathway. (**B**) The mitochondrial RCR level in liver tissues of mice in in NC, NE, HFD, HFE groups. *P < 0.05, **P < 0.01.

Based on the above results that Glucagon signaling pathway was significantly activated in HFE group, we analyzed the correlation of *Runx1t1* with genes significantly enriched in Glucagon signaling pathway. In HFD group, the *Runx1t1* expression exhibited an observable negative correlation with *Acaca* (Fig. 6A). Subsequently, we explored the mitochondrial RCR in NC, NE, HFD, HFE groups. Comparing to NC group, the mitochondrial RCR level in liver tissues was significantly decreased in HFD group (Fig. 6B). The mitochondrial RCR level was observably increased in HFE group than that in HFD group (Fig. 6B). The results indicated that endurance exercise could improve mitochondrial respiratory dysfunction in obese mice.

## Discussion

Obesity is a multifactorial disease with increasing burden and incidence worldwide. Moreover, obesity is often complicated by some other diseases, such as cardiovascular diseases^21^, type 2 diabetes mellitus (T2DM)^22^, hypertension^23^ and so on. Previous studies have reported that endurance training and endurance strength training significantly decreased the body mass, total body fat mass, waist and hip circumference of obese patients^24^. In this study, our results exhibited that endurance exercise had no significant effect on body weight but remarkably reduced the BFI of HFD-induced obese mice. Donnelly et al. conducted two 18-month training programs (one continuous and the other intermittent) in 22 untrained obese women, none of which induced clinically significant weight loss^25^. In addition, Kraus et al. found that the highest amount of weekly exercise could improve the lipidemic profile of 58 sedentary overweight people, but had minimal weight change^26^. These suggested that regular exercise or endurance exercise could alleviate the BFI of obesity in the absence of body weight loss. Thus, we investigated the target genes related to the improvement of BFI by endurance exercise in HFD-induced obese mice.

Firstly, we identified 133 genes associated with endurance exercise improving BFI in obese mice, and 16 genes were significantly enriched in 13 KEGG signaling pathways among these 133 genes. In addition, of these 16 genes, *Runx1t1* was a transcription factor gene and it was involved in the negative regulation of adipocyte differentiation^20^. Runt-related transcription factor 1 translocation partner 1 (*Runx1t1*), also known as *ETO*, *MTG8, CBFA2T1*, is a member of the myeloid translocation gene family^27^. *Runx1t1* mRNA could be detected in many tissues, such as brain, heart, skeletal muscle and adipose tissue^28,29^, *Runx1t1* can be expressed in two isoforms, a long (*Runx1t1-L*) and a short (*Runx1t1-S*)^30^. *Runx1t1* was firstly noted for its role in neurogenesis^31^. In addition, *Runx1-Runx1t1* has been reported to be associated with hematopoiesis and acute myeloid leukemia^32,33^. *Runx1t1* is also involved in the adipogenesis. In both 3T3-L1 preadipocytes undergoing differentiation and the mature adipocytes of rat, the *Runx1t1* mRNA expression was remarkably lower^20^, which was consistent with our results that the mRNA and protein levels of Runx1t1 decreased in HFD group compared to HFE, NC and NE groups. CCAAT-enhancer-binding proteins (C/EBP) and peroxisome proliferator-activated receptor γ (PPARγ) were major transcription factors of adipogenesis^34^. It has been reported that *Runx1t1* might inhibit the adipogenesis in 3T3-L1 cells through downregulating the expression of C/EBPα by inhibiting the C/EBP activity as well as downregulating the PPARγ expression^20^. Deng et al. have indicated that the *Runx1t1* expression was decreased first, and then subsequently increased during ovine preadipocyte differentiation, and knockdown of *Runx1t1-L* could promote the lipid accumulation and preadipocyte differentiation in ovine preadipocytes^30^. In addition, Zhao et al. reported that fat mass and obesity-associated protein (FTO) could control the expression of *Runx1t1* by modulating N6-methyladenosine levels around splice sites, and thereby affected differentiation of adipogenesis^35^. Collectively, *Runx1t1* might be a target for endurance exercise to improve BFI in obese mice.

Moreover, the GSEA results showed that the mitochondrial function related pathway, glucagon signaling pathway^36^, was significantly activated in the HFE group compared to HFD group. Subsequently, we analyzed the correlation of *Runx1t1* with enriched genes in the glucagon signaling pathway, and found that the *Runx1t1* expression exhibited an observably negative correlation with *Acaca* in HFD mice. Considering the correlation of insulin signaling pathway one mitochondrial dysfunction. We hypothesized that *Runxlt1* might play an important role in HFD-induced mitochondrial dysfunction in obese mice. It has been reported that obesity was correlated with the adipocyte mitochondrial dysfunction^37^. Excessive nutrient intake could increase the free fatty acid concentrations, hyperglycemia and mitochondrial reactive oxygen species (ROS) production in adipocytes, causing mitochondrial dysfunction^38^. In mice, the loss of essential complex IV components (such as Cox5b) could cause age-dependent mitochondrial complex IV dysfunction, thereby decreasing fatty acid oxidation, increasing lipid accumulation and obesity^39^. In obese individuals, the mitochondrial oxidative capacity and mitochondrial biogenesis were reduced^40^, and the decreased mitochondrial biogenesis in obesity was correlated with inflammation, metabolic alterations and insulin resistance^41^. In addition, Jheng et al. found that the smaller and shorter mitochondria and incremental fission of mitochondria in the skeletal muscle of mice with genetic obesity and those with diet-induced obesity^42^. These evidences suggested that the mitochondrial dysfunction was associated with reduced mitochondrial oxidative capacity and biogenesis in obesity. In present study, the mitochondrial RCR level was observably increased in HFE mice than that in HFD mice. It has been demonstrated that exercise training can alleviate the mitochondrial dysfunction, maintain the balance between mitochondrial dynamics and mitophagy, and reduce the apoptotic signaling in obese skeletal muscle^43^. In addition, compared with HFD mice, the *Runx1t1* mRNA and protein levels in liver tissues increased in HFE mice. Our data suggested that endurance exercise might improve mitochondrial dysfunction in HFD-induced obese mice by activating the *Runx1t1*. Nevertheless, to elucidate the mechanisms underlying endurance exercise improving mitochondrial dysfunction, more investigation should be performed in our future study.

## Conclusions

In summary, our work has revealed that *Runx1t1* is implicated in the improvement of BFI by endurance exercise in HFD-induced obese mice for the first time. Endurance exercise is able to improve mitochondrial dysfunction in HFD-induced obese mice by activating the *Runx1t1*. Our findings provide valuable information for understanding the molecular mechanism by which endurance exercise improves BFI or lipid metabolism in obese mice.

### Supplementary Information

Supplementary Figure 1.

Supplementary Table 2.

Supplementary Table 3.

Supplementary Table 4.

## Data Availability

The datasets generated and/or analyzed during the current study are available in the [NCBI SAR] repository, [WEB LINK: https://www.ncbi.nlm.nih.gov/bioproject/PRJNA980392, ACCESSION NUMBER: PRJNA980392].
