# Supplementary material for: Hepatic Runx1t1 improves body fat index after endurance exercise in obese mice
Source: Sci Rep. 2023 Nov 8;13:19427. doi: 10.1038/s41598-023-46302-w (PMC10632374; doi:10.1038/s41598-023-46302-w)
Supplement: Supplementary file 1 — Supplementary Figure 1. [file 41598_2023_46302_MOESM1_ESM.pdf]

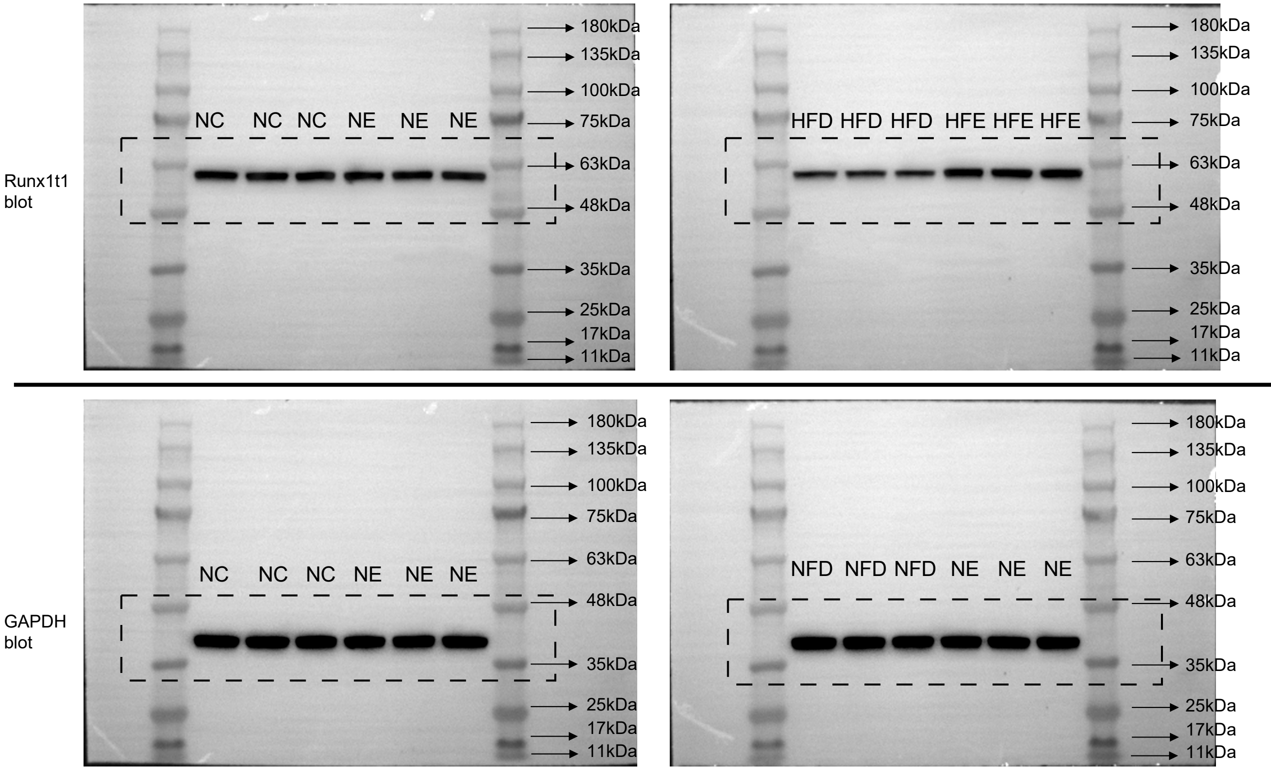

Note: The dashed line section is the final cropped image, and SDS-PAGE electrophoresis was configured with separating gel (8%-15%) and concentrating gel (5%).
